# Lineage Divergence of *Dendrolimus punctatus* in Southern China Based on Mitochondrial Genome

**DOI:** 10.3389/fgene.2020.00065

**Published:** 2020-02-19

**Authors:** Huicong Du, Man Liu, Sufang Zhang, Fu Liu, Zhen Zhang, Xiangbo Kong

**Affiliations:** ^1^Key Laboratory of Forest Protection of National Forestry and Grassland Administration of China, Research Institute of Forest Ecology, Environment and Protection, Chinese Academy of Forestry, Beijing, China; ^2^Guizhou Institute of Biology, Guizhou Academy of Sciences, Guiyang, China

**Keywords:** *Dendrolimus punctatus*, *Pinus massoniana*, divergence pattern, population, genetic structure

## Abstract

In southern China, the masson pine caterpillar, *Dendrolimus punctatus*, has caused serious damage to the *Pinus massoniana* (Lamb.) pine forests. Here, the whole mitochondrial DNA (mtDNA) was employed to analyze the population evolution of *D. punctatus* and to understand the process underlying its current phylogenetic pattern. *D. punctatus* populations within its distribution range in China were categorized into five subgroups: central and eastern China (CEC), southwestern China (SWC), Yibin in Sichuan (SC), Baise in Guangxi (GX), and Luoding in Guangdong (GD), with a high level of haplotype diversity and nucleotide diversity among them. The genetic distances between subgroups are relatively large; however, the genetic distances between populations within the CEC subgroup were relatively small, suggesting that many populations were closely related in this subgroup. The mantel test showed that geographic distance had an important impact on the genetic distance of different geographic populations (*r* = 0.3633, *P* < 0.001). The neutrality tests, Bayesian skyline plot, and haplotype network showed that *D. punctatus* experienced a population expansion around 100,000 years ago. The divergence times of GX/SC, SWC, GD, and CEC were 0.347, 0.236, 0.200, and 0.110 million years ago, respectively. The SWC, CEC, and GD subgroups might have evolved from GX or SC subgroups. The population genetic structure of *D. punctatus* was closely related to its host tree species, geographic distance among populations, the weak flight capacity, and many eco-environment conditions.

## Introduction

The masson pine caterpillar moth, *Dendrolimus punctatus* Walker (Lepidoptera: Lasiocampidae), is the most destructive insect pest among the >27 pine caterpillar species in China, causing tremendous economic damage annually to the pine forests, especially the *P. massoniana* Lamb. forests in southern China. *D. punctatus* is mostly distributed in the provinces south of the Qinling Mountains-Huai River line, and it has multiple generations per year that vary geographically. For example, in Xinyang district of Henan province, *D. punctatus* has two generations per year, but in Guangdong province, 3~4 generations occur (Xiao et al., 1964). The moth larvae often feed on conifer needles from February through October depending on their populations, with frequent widespread outbreaks occurring every 4~5 years, During a severe outbreak, an entire pine forest can be completely defoliated by *D. punctatus* larvae within several days, creating a scene of so called “smokeless forest fire” (Xiao et al., 1964; Zhang et al., 2004a). The frequent outbreaks of *D. punctatus* have led to extensive application of synthetic pyrethroids or other naturally derived insecticidal compounds, which has resulted in severe negative effects on biodiversity and the natural predators and parasitoids in the ecosystem (Wang et al., 2008). The resistance of *D. punctatus* to pyrethroids has been increasingly developed over the years (Li, 1991; Peng and Li, 1992). In addition, the geographic variation in the proportions/ratios of sex pheromone components in *D. punctatus* have clearly been demonstrated (Kong et al., 2006; Sun et al., 2017). Therefore, it is necessary and beneficial to study the evolutionary trends of different *D. punctatus* populations in order to identify their distribution characteristics and to develop efficient pest control measures targeting different populations of this economically important insect pest.

Recent single-species phylogeographic studies have demonstrated the strong power of mitochondrial DNA (mtDNA) data for resolving the divergence patterns and histories of geographic populations (Morin et al., 2010; Ma et al., 2012; Hirase et al., 2016; Fields et al., 2018), which is characterized by maternal inheritance, simple structure, and a rapid evolution rate. In recent years, mtDNA has been extensively employed in research on insect population heredity and estimated species pedigree history (Cameron et al., 2007; Du et al., 2019). The phylogeography of *D*. *punctatus* has been studied (Zhang et al., 2004a; Zhang et al., 2004b) and south China was considered to have acted as a refuge and a key distribution area for many remnant rare *Dendrolimus* species during the Quaternary glaciation. Based on the analyses of *D. punctatus* geohistory and its hosts, Zhang et al. (2004) concluded that the repeated climate changes in the Tertiary, Quaternary, and Interglacial periods strongly affected the population differentiation and distribution of *D. punctatus*. The genetic diversity and differentiation of *D. punctatus* populations in the fragmented habitats of Thousand Island Lake in Zhejiang province have been observed by Lv et al. (2018) using mitochondrial *COI* gene sequence analysis. In addition, *D. kikuchii*, which is closely related to *D. punctatus*, showed a high genetic diversity and a strong genetic differentiation among different populations, which were identified by *COI*, *COII*, and *Cyt b* markers (Men et al., 2017). However, studies on the population evolution of *D. punctatus* based on molecular data have been scarce over the last few decades.

Whole mtDNA sequences have much higher resolutions than partial mitochondrial gene sequences. Therefore, in this study, we used the whole mtDNA to explore the population evolution and genetic diversity as well as the differentiations of 48 individuals from 15 populations of *D. punctatus* collected from its main distribution regions in southern China. Additionally, we inferred the historical population dynamics of *D*. *punctatus* and discussed its geographic origin, population evolution trend, and biological characteristics, which are significant factors underlying the genetic variation of *D*. *punctatus* in China.

## Materials and Methods

### Insect

The cocoons of *D. punctatus* were collected from the main host, *Pinus massoniana* trees at 15 locations from nine provinces in 2016 (**Table S1**) and shipped to our Lab in Beijing. A total of 48 specimens of newly emerged adults were kept individually in absolute ethanol and stored in a −20°C freezer in the laboratory until DNA extraction.

### Mitogenome *De Novo* Sequencing and Assembly

Total genomic DNA was extracted from thoracic and leg muscle tissues using a Wizard Genomic DNA Purficication Kit (Qiagen, USA) following the manufacturer’s protocol. NanoDrop 2000 (Thermo Scientific, Wilmington, DE, USA) and picogreen (Thermo Scientific, Wilmington, DE, USA) were used to detect the DNA purity and concentration. To assess the DNA integrity, 1% agarose gel electrophoresis was used.

Genomic DNA were fragmented with Covaris M220 (Woburn, MA) to 400–500 bp. A 460-bp paired-end library was constructed from each specimen and sequenced to obtain 3 Gb of data using an Illumina Hiseq X Ten platform at Shanghai Majorbio Bio-pharm Technology Co., Ltd. Raw data were sheared as follows in order to make the subsequent assembly more accurate: the adapter sequence in reads was removed; the 5′-terminal non-AGCT base was detached; the ends of reads with low sequencing quality (< Q20) were removed; reads with an N ratio of 10% and small fragments with a length <25 bp were removed. All the downstream analyses were based on clean data of high quality (avg. Q20: 98.08%; avg. Q30: 94.35%, Mt genome sequencing coverage: 1648×). The clean data were assembled using SOAPdenovo v2.04 (http://soap.genomics.org.cn) (Luo et al., 2012; Xie et al., 2014), kmer was selected as 27, 29, 31, 33, 35, 37, 39, 41, 43, 45, 47, 49, 51, 53, 55, 57, and 59 respectively for splicing, and selected the optimal kmer as the final splicing result. Then, all the assembled contigs were compared with the Nr mitochondrial database and the compared contigs were screened out. MITObim v1.6 (Hahn et al., 2013) iterative alignment was used to map all sequenced clean reads onto the contig and the complete mitochondrial genome sequence was obtained by closing the gaps. The DOGMA (http://dogma.ccbb.utexas.edu) (Wyman et al., 2004) and MITO WebSever (Bernt et al., 2013) were used to predict the protein-coding genes (PCGs) from the mtDNA. PCGs was identified by alignment with homologous gene of the *D. kikuchii* (NC_036347.1). The complete mitochondrial genome sequences of *D. punctatus* were submitted to the GenBank, and accession numbers were listed in the **Table S1**.

### SNPs Analysis Based on mtDNA and Structure Analysis

The clean data after quality controlled were mapped to the reference genome (NC_036347.1) using BWA software (Li and Durbin, 2009), and then sequenced fragments generated on PCR duplication were removed using Picard-tools (https://github.com/broadinstitute/picard). The results were calibrated using GATK (https://github.com/broadinstitute/gatk) (Mckenna et al., 2014). SNP Pdetection was conducted using VarScan (https://github.com/dkoboldt/varscan) to filter out the low sequencing depth and low alignment quality of site, and SNP data sets with high credibility were obtained. The filtering conditions: minimum coverage depth: 30 reads; the minimum mutation frequency: 20%; minimum mutant base number: 15 reads; minimum mass value of mutation detection site: Q20; both forward and reverse reads needed to support mutant sites, and the number of forward and reverse reads varied by less than 10%. The SNPs obtained by combining the data with gff gene annotation information on reference species were annotated using the Annovar program (http://www.openbioinformatics.org/annovar/annovar_gene.html) (Yang and Wang, 2015). Population structure analysis was performed using STRUCTURE v2.3.4 (Pritchard et al., 2000). To determine the most likely group number, STRUCTURE was run ten times for each K value from 2 to 8. The length of the burn-in period was 200,000. The number of Markov chain Monte Carlo (MCMC) reps after burn-in was set to 1,200,000. The optimal K value of 5 was selected using the Evanno method (Evanno et al., 2005).

### Genetic Diversity, Genetic Distance and Isolation by Distance (IBD)

The sequences were preliminarily aligned using the Clustal X program (Thompson et al., 1997). The number of haplotypes, haplotype diversity (Hd), nucleotide diversity (Pi), and mean number of nucleotide differences (K) in each population were calculated using DnaSP v5.0 (Librado and Rozas, 2009). The pairwise genetic distances were calculated using MEGA v6.0 based on the Kimura-2-parameter model (Kimura, 1980; Tamura et al., 2013). We applied multiple methods to explore the demographic history of different lineages including the neutral test using Tajima’s D (Tajima, 1989) and Fu’s Fs (Fu, 1997) implemented in Arlequin. Based on longitude and latitude, we calculated the geographic distance of the sampling sites. The haplotype network of *D*. *punctatus* was analyzed using a median-joining algorithm in the program Network4.6 (Bandelt et al., 1999). The Mantel test of IBD 1.53 (Bohonak, 2002) was used to assess the correlations between the genetic distance and the geographic distance of the populations. Furthermore, the historical population dynamics of the 15 locations were investigated using Bayesian skyline plot (BSP) analysis implemented in BEAST v1.7. (Drummond et al., 2005; Drummond et al., 2012).

### Phylogenetic Analysis

Forty-eight whole mtDNA sequences were aligned using the Clustal X program, and phylogenetic trees were constructed using the Bayesian inference (BI) and maximum likelihood (ML) methods after the correction. MrMTgui software (Nylander, 2004) was used to compare and analyze the alternative models of sequences, and the optimal alternative model (GTR+G) was used to construct the evolutionary tree. The Bayesian tree was obtained by running MrBayes v3.1.2 (Ronquist and Huelsenbeck, 2003). The MCMC method was used to calculate 3 million generations, sampling once every 100 generations to ensure independence of sampling. The original 3,000 trees were discarded as burn-in. We constructed phylogenetic trees (ML) based on the mtDNA and SNPs by MEGA 7.0.26, and the confidence values of the ML tree were calculated through the bootstrap test using 1,000 iterations (Guindon and Gascuel, 2003).

### Estimation of Divergence Time

Based on the 13 protein-coding genes of *D*. *punctatus*, we performed divergence time estimation for different populations using BEAST v1.10.4 (Bouckaert et al., 2014). MrMTgui software was used to select the most suitable sequence replacement model (GTR) for comparative analysis. The Heterogeneity Model was set to Gamma + Invariant Sites; Clocks was set to Strict Clock; Tree Prior was set to Speciation. The Yule process calculated 100,000,000 generations with the MCMC method, by sampling once every 1,000 generations and discarding the first 10% as burn-in. Due to the lack of a suitable fossil record as a time marker, we used the published molecular clock of protein-coding genes in insect mitochondrial genomes, the mean rate of *COX1* was set as 0.0177 substitutions/site/Myr (s/s/Myr) (Papadopoulou et al., 2010), The substitution rates of other mitochondrial genes were scaled to the *COX1* mean rate and weak lognormal distributions with large standard deviations were applied to allow the *COX1* calibration to control the analysis. Finally, Tracer v1.5 (http://tree.bio.ed.ac.uk/software/tracer/) and FigTree v1.3.1 (http://tree.bio.ed.ac.uk/software/figtree) were used to calculate and display the results.

## Results

### Genetic Diversity and Haplotype Composition of *Dendrolimus Punctatus*

The number of segregating sites, haplotype diversity, nucleotide diversity, and mean number of nucleotide differences in *D*. *punctatus* among different populations were 892, 1, 0.0107, and 125.8, respectively (**Table 1**). The genetic diversity index of each population was somewhat different. Except for HNDA, JXYD, and GXBS populations, the index of nucleotide diversity and mean number of nucleotide differences in the left populations were less than the corresponding values of the total populations. The mtDNA data of *D*. *punctatus* from all populations were tested using neutrality tests method. The results showed that Tajima’s D value and Fu’s Fs value were both negative, indicating that the *D*. *punctatus* populations had experienced a population expansion in the past.

**Table 1:** Haplotype and nucleotide diversity analysis, and neutrality tests based on mitochondrial DNA (mtDNA) of 15 populations of *Dendrolimus punctatus*.

Province code	Population code	S	Hd	Pi	k	Tajima’s D	Fu’s Fs
HB	HBDW	89	1	0.0051	59.3	−0.063 (P > 0.10)	−0.645(P > 0.10)
	HBHG	71	1	0.0071	47.7		
HN	HNCZ	60	1	0.0034	40	−0.874 (P > 0.10)	−0.961 (P > 0.10)
	HNDA	190	1	0.0108	127		
SC	SCDZ	31	1	0.0017	20.7	0.958(P > 0.10)	0.741 (P > 0.10)
	SCYB	62	1	0.0036	41.7		
CQ	CQYB	156	1	0.0089	104	−1.060 (P > 0.10)	−1.185 (P > 0.10)
	CQNC	61	1	0.0035	40.7		
JX	JXYD	208	1	0.0118	138.7	−0.943 (P > 0.10)	−1.039 (P > 0.10)
	JXXG	94	1	0.0054	62.7		
GX	GXBS	224	1	0.0127	149.3	0.157 (P > 0.10)	0.161 (P > 0.10)
	GXGL	71	1	0.0041	47.7		
GD	GDLD	45	1	0.0021	24.8	−0.710 (P > 0.10)	0.018 (P > 0.10)
GZ	GZJP	196	1	0.0096	112	0.333 (P > 0.10)	0.369 (P > 0.10)
AH	AHHF	78	1	0.0036	42	−0.121(P > 0.10)	−0.106 (P > 0.10)
Total		892	1	0.0107	125.8	−1.496 (P > 0.10)	−2.209 (0.05 < P < 0.10)

This table includes number of polymorphic sites (S), haplotype diverpsity (Hd), nucleotide diversity (Pi), mean number of nucleotide differences (k), Tajima’s D and Fu’s Fs.

**Figure 1:**
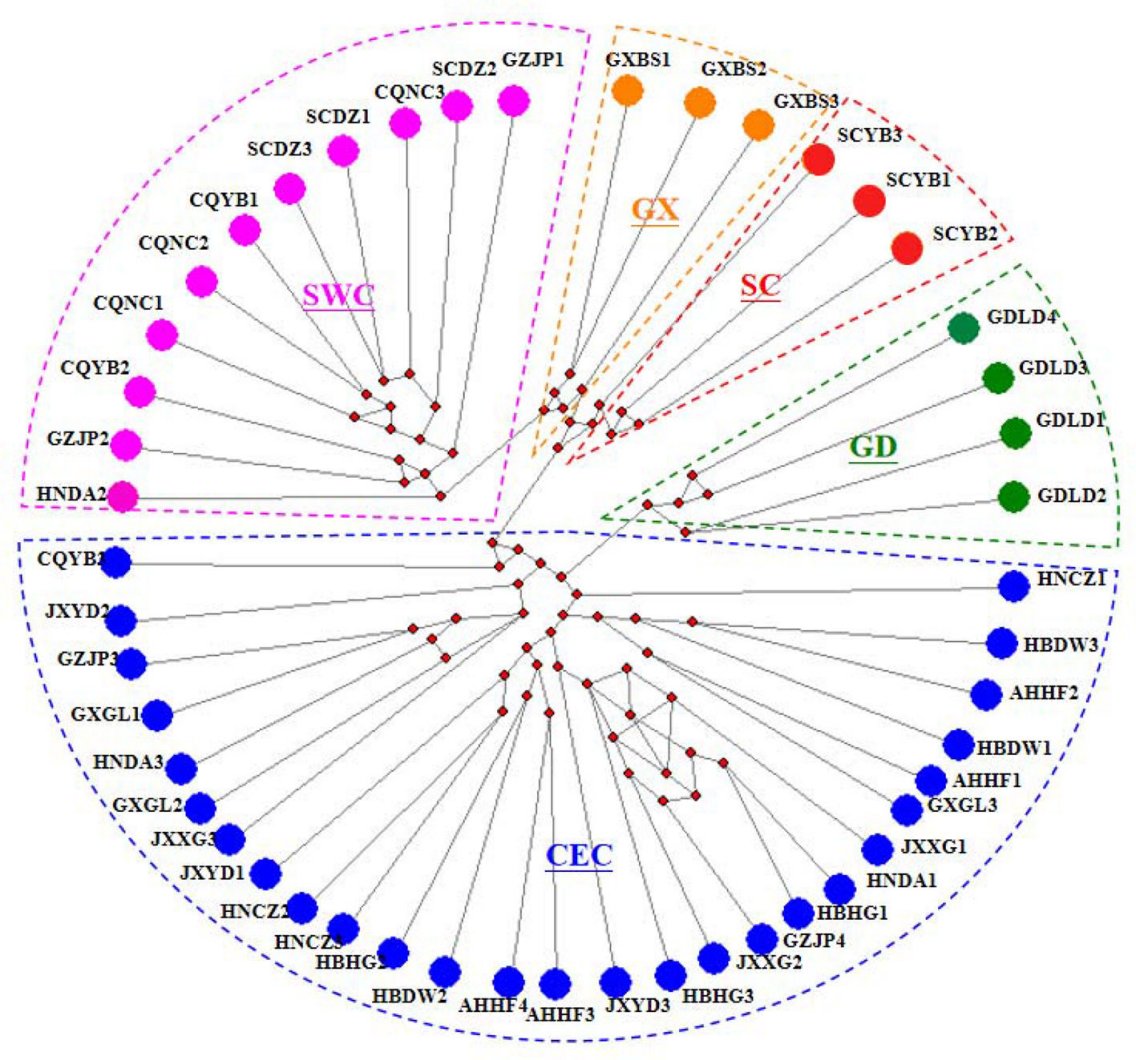
Median-Joining network based on the whole mitochondrial DNA (mtDNA). Each circle represents a haplotype and next to the haplotype is the populations to which the haplotype belongs. Different colored areas in the dotted line correspond to different subgroups. The little red dots in the middle of the circle represent the mutational nodes.

We performed a haplotype analysis based on the mitochondrial genome of *D*. *punctatus*, and obtained 48 haplotypes from 15 geographic populations, all of which were exclusively haplotypes. The haplotype network established based on the mitochondrial whole genome by using the Median-joining method produced a continuous network structure (**Figure 1**), which was generally stellate in shape, indicating that the pine caterpillar experienced a population expansion. The haplotype network diagram can divide the haplotypes into two multiple haplotype clades (Clade CEC and SWC) and three monotypic clades (Clades GX, SC, and GD).

### Population Structure and Phylogenetic Analyses Based on mtDNA and SNP

**Figure 2:**
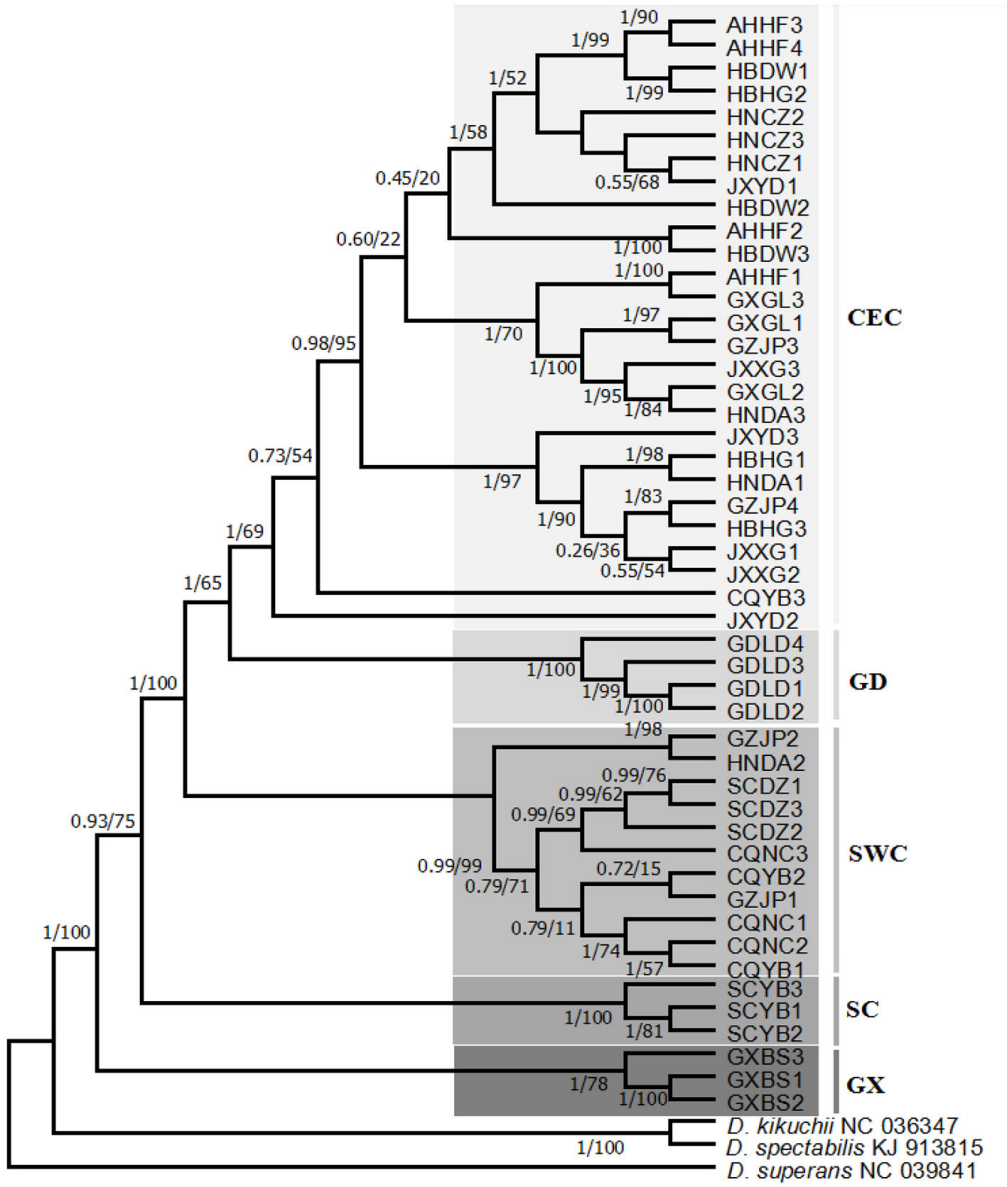
Phylogenetic tree [Bayesian inference (BI) and maximum likelihood (ML)] of 15 geographic populations based on the whole mitochondrial DNA (mtDNA) of *Dendrolimus punctatus*. Numbers above or below the branches indicate the bootstrap values, for BI/ML, respectively. Clades with different colors indicate different branches.

Phylogenetic trees (BI and ML) were constructed using the whole mtDNA data of 48 sequences of *D*. *punctatus* and combining them with data on *D*. *spectabilis*, *D*. *kikuchii*, and *D*. *superans* (**Figure 2**). In the phylogenetic tree, *D*. *punctatus* populations were clustered into five lineages: central and eastern China (CEC), Luoding in Guangdong (GD), southwestern China (SWC), Yibin in Sichuan province (SC), and Baise in Guangxi province (GX). Populations of Sichuan Yibin (SCYB), Guangxi Baise (GXBS), and Guangdong Luoding (GDLD) were separate lineages, and the monophyly of these three lineages was well supported; Sichuan Dazhou (SCDZ), Chongqing Nanchuan (CQNC), and Yubei (CQYB), plus part of Guizhou Jinping (GZJP) and Hunan Dongan (HNDA) were clustered in the SWC lineage; CEC lineage included the populations of Anhui Hefei (AHHF), Hubei Dawu (HBDW), Hubei Huanggang (HBHG), Hunan Chenzhou (HNCZ), Hunan Dongan (HNDA), Jiangxi Xingguo (JXXG), Jiangxi Yudu (JXYD), Guangxi Guilin (GXGL), Guizhou Jinping (GZJP) and plus one of CQYB. The phylogeny complied well with the results from the haplotype network. In the topological structure of phylogenetic tree, individuals in GXBS, SCYB, SCDZ, GDLD, and HNCZ clustered together by their separated populations, while individuals in other populations mixed and clustered together among populations, which may be related to certain gene exchanges between populations.

**Figure 3:**
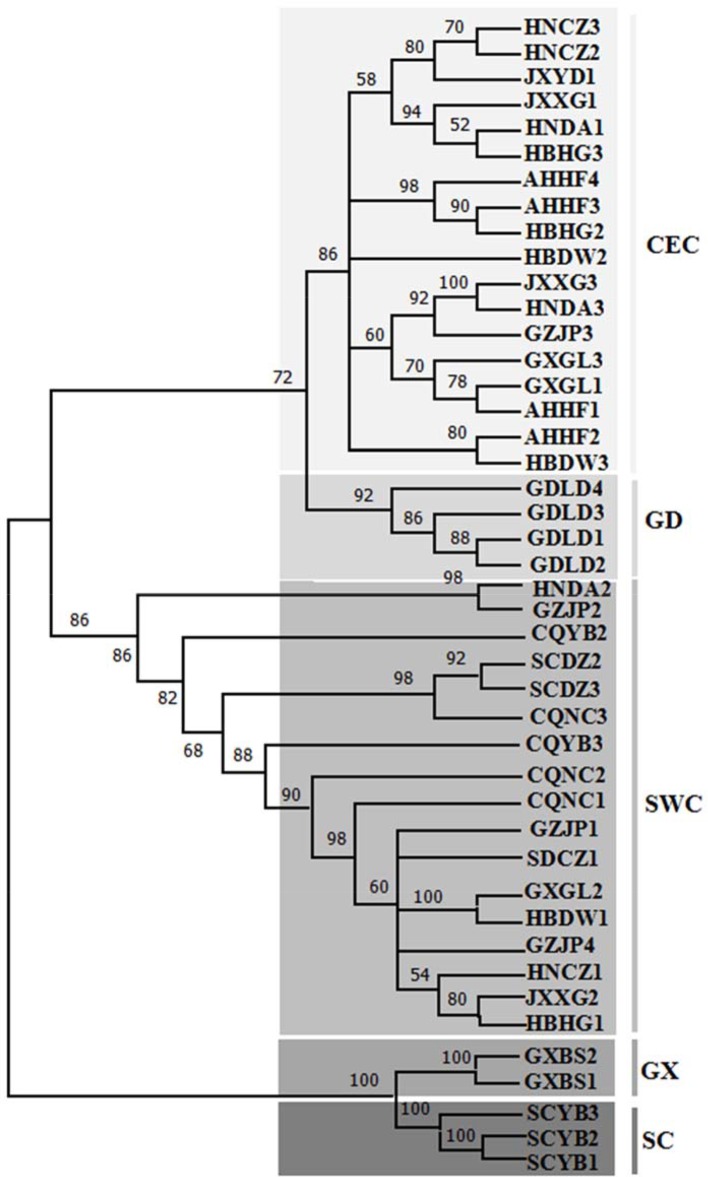
Phylogenetic tree [maximum likelihood (ML)] of 15 geographic populations based on SNP of the whole mitochondrial DNA (mtDNA) of *Dendrolimus punctatus*. Numbers above the branches indicate the bootstrap values. Clades with different colors indicate different branches.

The phylogenetic tree of 44 SNPS (**Figure 3**) of *D*. *punctatus* was constructed by maximum likelihood method, and the phylogenetic analysis of SNPs indicated that their phylogenetic relationships were similar to those of mitochondrial genome. The individuals in GDLD population were clustered into one lineage, two lineages of GD and CEC clustered together, and also there were a few individuals being different from the topology of phylogenetic trees based on mitcochondrial genomes, and five individuals of CEC subgroups clustered into the SWC group. The monophyly of the two lineages—the GX group and the SC groups—was well supported. The results of population structure construction based on SNPs showed that the most reasonable sample classification method was at K = 5 (**Figure S1**). Therefore, the five lineages represented the most likely evolutionary development and diversification of *D*. *punctatus*.

### Genetic Distance and IBD Test

The pairwise genetic distances ranged from 0.004 to 0.020 (**Table 2**). Of the 105 comparisons, genetic distances between 65 pairs were more than 0.01. All the populations of GXBS, GDLD, and SCYB had genetic distance values >0.01 between each other and from other populations, indicating that the genetic distances between these three populations and other populations were far higher. In contrast, the genetic distance values between any two populations of GXGL, AHHF, GZJP, HBDW, HBHG, HNCZ, HNDA, JXXG, and JXYD were lower (< 0.01), suggesting that these populations are genetically closer (**Table 2**). All or parts of these nine populations belonged to the CEC group, which corresponded nicely to the phylogenetic tree.

**Table 2:** Pairwise genetic distances based on whole mitochondrial DNA (mtDNA) data from 15 populations of *Dendrolimus punctatus*.

Population code	AHHF	CQNC	CQYB	GDLD	GXBS	GXGL	GZJP	HBDW	HBHG	HNCZ	HNDA	JXXG	JXYD	SCDZ
AHHF														
CQNC	0.011													
CQYB	0.010	0.006												
GDLD	0.010	0.012	0.012											
GXBS	0.018	0.019	0.019	0.018										
GXGL	0.004	0.011	0.010	0.010	0.018									
GZJP	0.008	0.008	0.009	0.011	0.019	0.008								
HBDW	0.004	0.011	0.010	0.010	0.018	0.005	0.008							
HBHG	0.004	0.012	0.011	0.010	0.018	0.005	0.008	0.005						
HNCZ	0.004	0.011	0.010	0.010	0.018	0.005	0.008	0.004	0.004					
HNDA	0.008	0.010	0.010	0.012	0.020	0.008	0.009	0.008	0.008	0.008				
JXXG	0.005	0.012	0.011	0.010	0.018	0.005	0.008	0.006	0.005	0.005	0.008			
JXYD	0.008	0.013	0.012	0.013	0.020	0.009	0.010	0.009	0.008	0.008	0.011	0.009		
SCDZ	0.011	0.004	0.007	0.012	0.019	0.012	0.008	0.012	0.012	0.012	0.011	0.012	0.013	
SCYB	0.017	0.018	0.017	0.018	0.020	0.017	0.017	0.017	0.017	0.017	0.018	0.018	0.019	0.018

The results of the Mantel test showed that there were significant correlations between the genetic distance and geographic distance. The test results had *r* = 0.3633 (*P* < 0.001), suggesting that the genetic distance and genetic differentiation among *D*. *punctatus* populations are related to their geographic isolation.

### Estimation of Divergence Time

**Figure 4:**
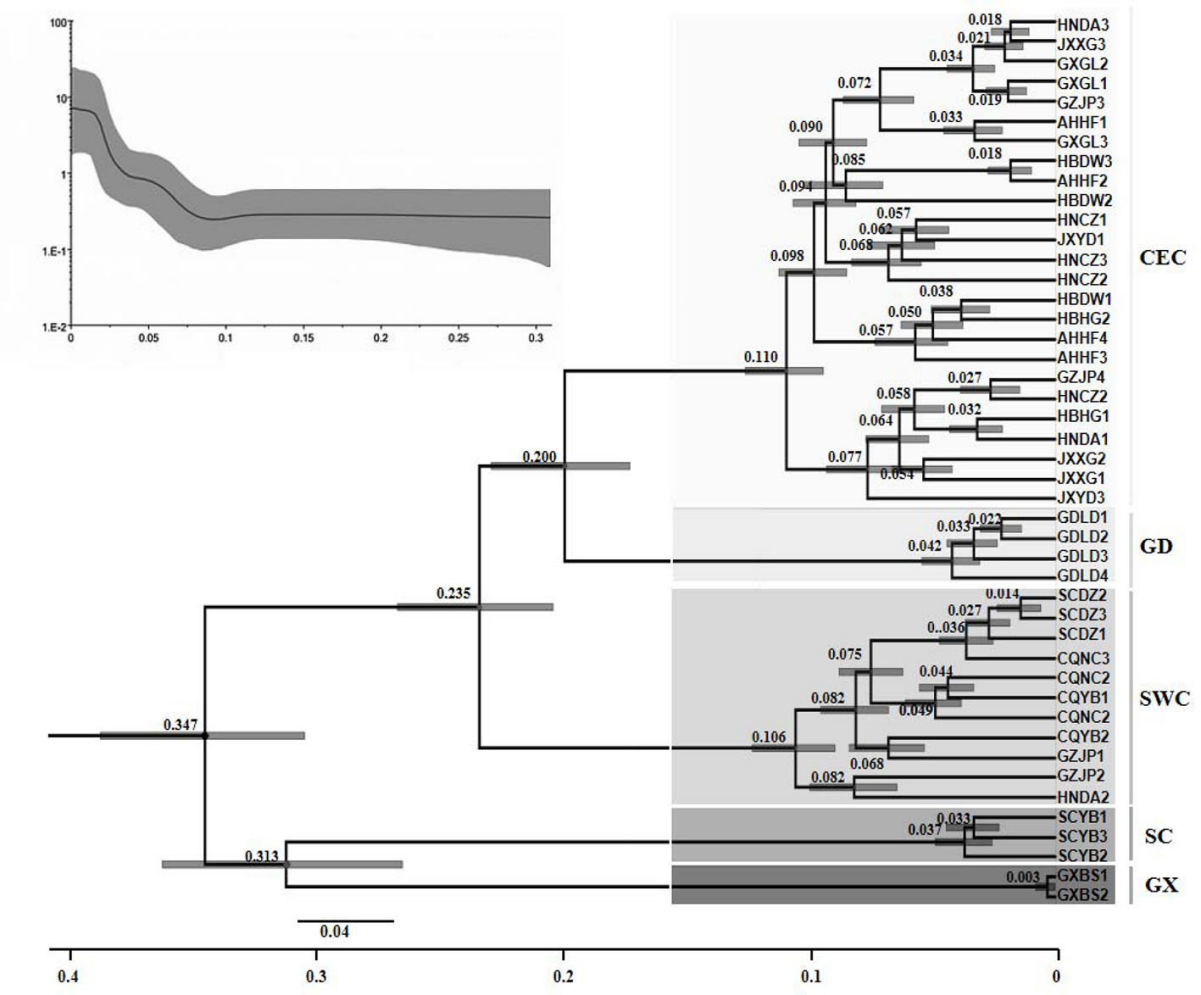
Divergence time tree of the populations of *Dendrolimus punctatus*. Numbers above or below branches indicate the divergence time (in million years) of the branches (Lower trace). Historical dynamics of the populations of *Dendrolimus punctatus* [Bayesian skyline plot (BSP) (upper trace)]. The middle solid line indicates the mean population size, and the gray area below and above the solid line indicates the 95% confidence interval.

The phylogenetic analysis of different *D*. *punctatus* geographic populations showed that the divergence time of SC and GX lineages was 347,000 years ago (95% highest posterior density interval: 305,800~389,200), which was the earliest divergence time among all five lineages (**Figure 4**). The SWC lineage diverged 235,000 years ago, the GD lineage was formed 200,000 years ago, and the divergence time of the CEC lineage was 110,000 years ago. Most of the divergence times related to population differentiation were within 100,000 years, which is consistent with the results obtained by BSP analysis.

Furthermore, the BSP analysis showed that the populations of *D*. *punctatus* have been maintained in a stable state for a long period of time (**Figure 4**). The analysis indicated that the *D*. *punctatus* populations began to expand rapidly 100,000 years ago and the population growths increased rapidly, confirming the fact that *D*. *punctatus* experienced population expansions in the past.

## Discussion

The Chinese masson pine caterpillar, *D*. *punctatus*, poses a huge threat to the economy and landscape related to the pine forests in southern China as it seriously harms the growths of pine trees and reduces the resin productivity. The mitochondrial genome as the molecular marker was employed to study the population lineage divergence of *D*. *punctatus* sampled throughout its main distribution areas in China in this paper. All the studied specimens were collected from different sites in each population in order to avoid collecting siblings. Finally, 48 mitochondrial genome sequences of *D*. *punctatus* from 15 locations in southern China were obtained, and all of these sequences shared the same gene arrangements as *Drosophila yakuba* (Clary et al., 1982). Our results showed that the nucleotide diversity (Hd) of *D*. *punctatus* populations was 0.0107, similar to the results of an individual population obtained *via* the *Cyt b* gene analysis (Pi = 0.0145) (Gao et al., 2008) with a high-level haplotype diversity and genetic diversity. The existence of exclusive haplotypes indicates that different geographic populations exhibit high genetic differentiations.

Structure and phylogenetic analysis of whole mtDNA indicated that *D*. *punctatus* populations were geographically structured in five lineages: populations of central and eastern China (CEC), southwestern China (SWC), Yibin in Sichuan (SC), Baise in Guangxi (GX), and Luoding in Guangdong (GD). A few specimens belonging to the same population were classified into different subgroups but their clustering boundaries were not obvious, which might be related to the existence of a small amount of gene flow between the subgroups. The formation of these five subgroups may be related to the existence of certain natural geographic barriers between them. Wushan and Wuling Mountains are located at the border of Chongqing and Hubei, hindering the physical exchanges and interactions between the SWC and the CEC lineages. However, the genetic distance between them was small, indicating that the differentiation time due to isolation was not long ago. The Guangdong population locates in the southeast of China, and Wuyi and Nanling Mountains in the north may have blocked the exchanges between the GD lineage and the other lineages to some extent, and GD lineage occurred 3~4 generations more than other lineages, and this lineage might have formed a stable genetic structure, which was also the reason for being monophyletic lineage. The monophyly of SCYB and GXBS populations is also well supported, with mountains and distances between the two lineages blocking cold air from the north and the Indian Ocean, the gene exchanges between the two populations and other populations were also prevented.

**Figure 5:**
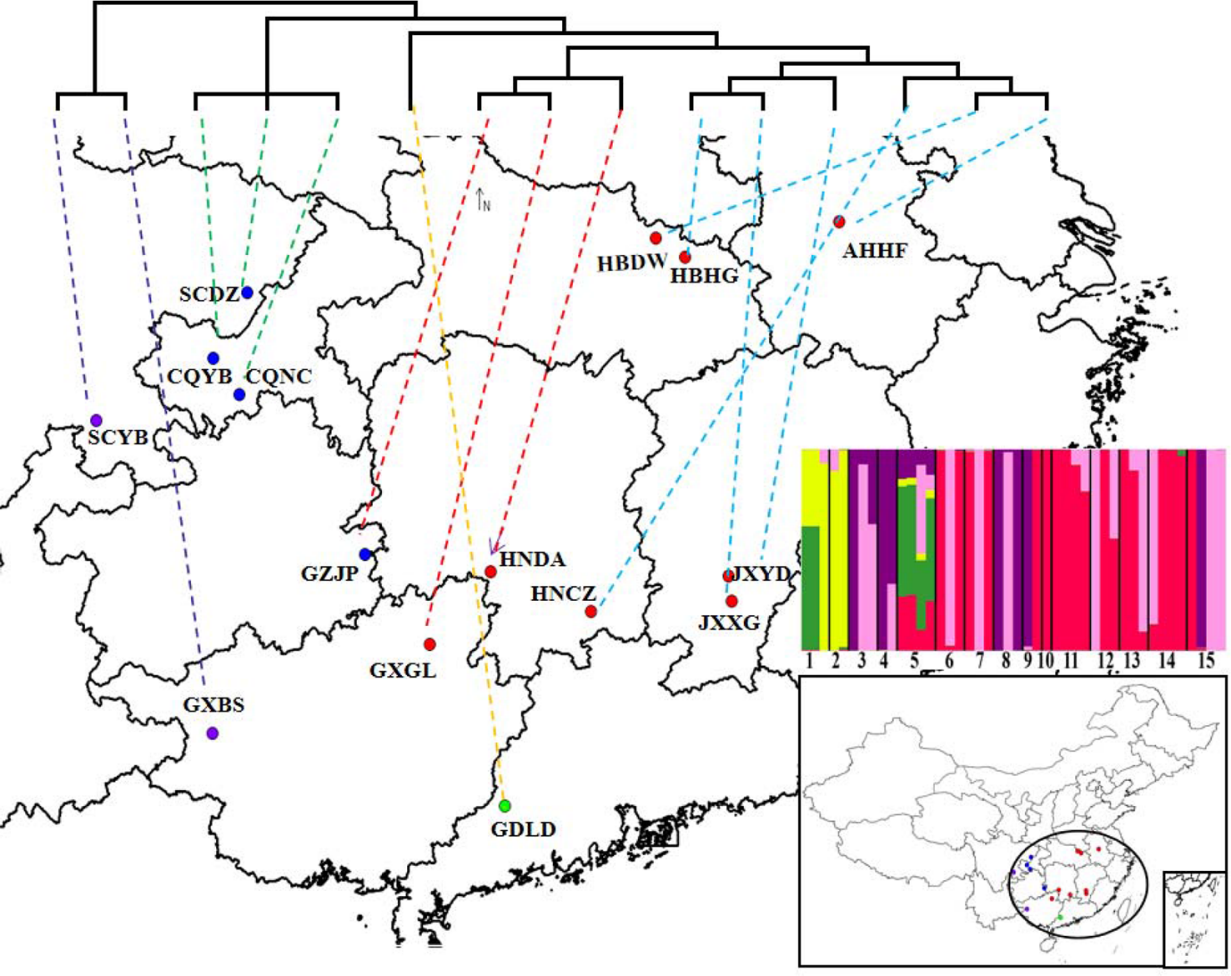
The locations of sampling specimens of *Dendrolimus punctatus*. A simplified maximum likelihood (ML) tree based on mtDNA is shown at the top of the figure. Lower-right insert shows the best genetic clustering (K = 5) based on SNPs of the whole mtDNA. The populations represented by numbers 1~15 in turn are: GXBS, SCYB, CQNC, CQYB, GDLD, HNCZ, HBDW, HNDA, SCDZ, AHHF, GXGL, HBHG, JXXG, JXYD, and GZJP.

In our study, the genetic distance results were consistent with the results of the phylogenetic tree clustering. The genetic distance between populations within the CEC subgroup is generally small, and these populations are closely related, which may be associated with the geographical characteristics of the subgroup, even though the CEC subgroup is the most widely distributed of the five subgroups. The majority of populations in the CEC subgroup are distributed in the middle and lower reaches of the Yangtze River in China (**Figure 5**), which are associated with few geographic barriers and frequent human activities, making the gene exchanges among the populations easier and their genetic distances smaller. The genetic distances were significantly and positively correlated with their geographic distances, suggesting that the geographic distance is one of the key factors affecting the genetic distances and relationships between *D*. *punctatus*’ populations. This is consistent with the results of a study by Men et al. (2017) on the population genetic differentiation of *D*. *kikuchii*.

In addition to the influence of distance and geographic barriers, population evolution and genetic diversity of a species are also affected by its own biological characteristics and eco-environmental conditions, such as habitat specialization, flight capacity, and population size (Sumner et al., 2004; Peakall and Lindenmayer, 2006; Fuentes-Contreras et al., 2008). Species with a high dispersal ability and a large number of individuals within a population have a great chance of maintaining gene flow and panmixia. In contrast, a low dispersal capacity and poor habitat availability will result in species subpopulations becoming isolated, with gene flow absent and genetic drift being independent within each subpopulation (Louy et al., 2007; Dixo et al., 2009). *D*. *punctatus* moths are generally considered as a sedentary species based on previous research on their flight capacity (Tong and He, 2009). As migration ability of the adults is weak and migration rarely occurs when food sources are sufficient, the phenomenon of rapid differentiation is accelerated. This is also the reason why the genetic distance among subgroups was greater than that among populations within subgroups.

All living creatures on earth are descendants of the survivors of the Quaternary ice age 2 million years ago. The Qinling Mountain and Yunnan-Guizhou plateau acted as barriers to slow down the glaciation and provided a favorable environment for the protection and migration of plants and animals. Based on the phylogenetic tree and differentiation time analysis, we speculated that *D*. *punctatus* originated in the GX and SC subgroups. The first divergence time corresponds to the Lushan glacial period (0.37–0.24 million years ago) of the Quaternary glacial period in China (Jing and Liu, 1999) and the Lushan-Dali interglacial period was 235,000 years ago, during which the continental climate was warm and *D*. *punctatus* began to spread from the Sichuan basin region to the east. Due to mountain barriers, *D*. *punctatus* expanded along the Yangtze River, first evolved into the SWC subgroup, and then further expanded along the Yangtze River system to areas in central and eastern China 200,000 years ago. Most populations of *D*. *punctatus* diverged between 100,000 and 10,000 years ago. During this time, the Chinese mainland was in the interglacial period, the climate was warm, and the *D*. *punctatus* populations exhibited increased migration, which gradually led to the current genetic distribution pattern of *D*. *punctatus*. Based on *COI*, Li et al. (2019) also estimated that the population expansion of *D*. *punctatus* started at about 0.23 million years ago. Xu et al. (2012) showed that the differentiation time of large-hoofed bats in China ranges from 85,700 to 91,600 years, which represents the same period as the population expansion of *D*. *punctatus*. In addition, *Myotis davidii* in the central and eastern plains also experienced a population expansion 79,000 years ago (You et al., 2010).

More previous studies on population variations implied that the diversification of hosts could have significant impacts on the differentiation of insects (Singer and Moore, 1991; Friberg et al., 2014; Friberg et al., 2016). *D*. *punctatus* is a typical phytophagous insect, and its population distribution patterns are strongly related to its host plants. The main host of *D*. *punctatus* in southern China is *P*. *massoniana*. According to a phylogeographic study, *P*. *massoniana* experienced a recent expansion of its smaller populations during the Quaternary period (Ge et al., 2012). Furthermore, recent studies have confirmed that *P*. *massoniana* also existed in multiple glacial refuges fostering populations with a high genetic diversity (Zhou et al., 2010; Ge et al., 2012), which is consistent with the situation that *D*. *punctatus* has a large genetic distance between subgroups. The populations of *D*. *punctatus* are thought to have moved to the south of the Yangtze River and even farther at some time during the Jurassic and Early Cretaceous periods (about 42,000–140,000 years ago), based on its host plants’ distribution (Zhang et al., 2004a). All these evidences indicate that the hosts of *D*. *punctatus* not only provide abundant food resources and habitat, but also have a great influence on its population divergence.

## Conclusions

The population genetic diversity and evolution of *D*. *punctatus* sampled throughout its main distribution range in China were studied by the whole mtDNA analyses. A high level of genetic diversity among *D*. *punctatus* populations was observed in these sampled areas. Based on SNPs, the 15 populations were divided into five subgroups, corresponding with the five lineages of the phylogenetic tree based on the whole mtDNA data. *D*. *punctatus* experienced a population expansion. The genetic distances between populations were related to their geographic distances. It is speculated that *D*. *punctatus* originated in the GX and SC subgroups, spreading from west to east and from north to south, and is now widely distributed in the area to the south of Yangtze River in China. There are some limitations to study population differentiation only through mtDNA markers, nuclear genomes are also an important way to study populations, and many conflicting geographic patterns between mitochondrial and nuclear genetic markers have been identified (Toews and Bresford, 2012). These genetic data not only provide us with an understanding of the population genetics of *D*. *punctatus*, but also provide guidance for developing more efficient pest control strategies. Our next step will be to combine mitochondrial and nuclear genes to further analyze and study the coordination of population differentiations.

## Data Availability Statement

The data in the article can be found in GenBank and related information is in **Table S1**.

## Author Contributions

XK and ZZ conceived and designed the study. XK, ML, and HD conducted the field work and wrote the manuscript. SZ and FL revised the manuscript. HD performed the data analyses. All authors read, commented, and agreed on the manuscript.

## Funding

This study was supported by the National Natural Science Foundation of China (31470654).

## Conflict of Interest

The authors declare that the research was conducted in the absence of any commercial or financial relationships that could be construed as a potential conflict of interest.

## Supplementary Material

Table S1 Sampling information of *Dendrolimus punctatus* in China.

Figure S1 Population structure branch optimum value analysis.

## References

[B1] Bandelt H. Forster P. Röhl A. (1999). Median-joining networks for inferring intraspecific phylogenies. Mol. Biol. Evol. 16, 37–48. 10.1093/oxfordjournals.molbev.a026036 10331250

[B2] Bernt M. Donath A. Jühling F. Externbrink F. Florentz C. Fritzsch G. (2013). MITOS: improved *de novo* metazoan mitochondrial genome annotation. Mol. Phylogenet. Evol. 69, 313–319. 10.1016/j.ympev.2012.08.023 22982435

[B3] Bohonak A. J. (2002). IBD (Isolation by Distance): a program for analyses of isolation by distance. J. Hered. 93, 153–154. 10.1093/jhered/93.2.153 12140277

[B4] Bouckaert R. Heled J. Kühnert D. Vaughan T. Wu C. H. Xie D. (2014). BEAST 2: A software platform for bayesian evolutionary analysis. PLoS Comput. Biol. 10, e1003537. 10.1371/journal.pcbi.1003537 24722319 PMC3985171

[B5] Cameron S. L. Lambkin C. L. Barker S. C. Whiting M. F. (2007). A mitochondrial genome phylogeny of Diptera: whole genome sequence data accurately resolve relationships over broad timescales with high precision. Syst. Entomol. 32, 40–59. 10.1111/j.1365-3113.2006.00355.x

[B6] Clary D. O. Goddard J. M. Martin S. C. Fauron C. M. Wolstenholme D. R. (1982). *Drosophila* mitochondrial DNA: a novel gene order. Nucleic Acids Res. 10, 6619–6637. 10.1093/nar/10.21.6619 6294611 PMC326953

[B7] Dixo M. Metzger J. P. Morgante J. S. Zamudi K. R. (2009). Habitat fragmentation reduces genetic diversity and connectivity among toad populations in the Brazilian Atlantic Coastal Forest. Biol. Conserv. 142, 1560–1569. 10.1016/j.biocon.2008.11.016

[B8] Drummond A. J. Rambaut A. Shapiro B. Pybus O. G. (2005). Bayesian coalescent inference of past population dynamics from molecular sequences. Mol. Biol. Evol. 22, 1185–1192. 10.1093/molbev/msi103 15703244

[B9] Drummond A. J. Suchard M. A. Xie D. Rambaut A. (2012). Bayesian phylogenetics with BEAUti and BEAST 1.7. Mol. Biol. Evol. 29, 1969–1973. 10.1093/molbev/mss075 22367748 PMC3408070

[B10] Du Z. Y. Hasegawa H. Cooley J. R. Simon C. Yoshimura J. Cai W. Z. (2019). Mitochondrial genomics reveals shared phylogeographic patterns and demographic history among three periodical cicada species groups. Mol. Biol. Evol. 36, 1187–1200. 10.1093/molbev/msz051 30850829 PMC6526903

[B11] Evanno G. Regnaut S. Goudet J. (2005). Detecting the number of clusters of individuals using the software STRUCTURE: a simulation study. Mol. Ecol. 14, 2611–2620. 10.1111/j.1365-294X.2005.02553.x 15969739

[B12] Fields P. D. Obbard D. J. McTaggart S. J. Galimov Y. Little T. J. Ebert D. (2018). Mitogenome phylogeographic analysis of a planktonic crustacean. Mol. Phylogenet. Evol. 129, 138–148. 10.1016/j.ympev.2018.06.028 29920335

[B13] Friberg M. Schwind C. Roark L. C. Raguso R. A. Thompson J. N. (2014). Floral scent contributes to interaction specificity in coevolving plants and their insect pollinators. J. Chem. Ecol. 40, 955–965. 10.1007/s10886-014-0497-y 25236381

[B14] Friberg M. Schwind C. Thompson J. N. (2016). Divergence in selection of host species and plant parts among populations of a phytophagous insect. Evol. Ecol. 30, 723–737. 10.1007/s10682-016-9835-6

[B15] Fu Y. X. (1997). Statistical tests of neutrality of mutations against population growth, hitchhiking and background selection. Genetics 147, 915–925. 10.0000/PMID9335623 9335623 PMC1208208

[B16] Fuentes-Contreras E. Espinoza J. L. Lavandero B. Ramirez C. C. (2008). Population genetic structure of codling moth (Lepidoptera: Tortricidae) from apple orchards in Central Chile. J. Econ. Entomol. 101, 190–198. 10.1093/jee/101.1.190 18330135

[B17] Gao B. J. Gao L. J. Hou J. H. Shang J. J. You L. Q. (2008). Genetic diversity of *Dendrolimus* (Lepidoptera) population from different geographic area. Acta Entomol. Sin. 28, 0842–0848. 10.3724/SP.J.1141.2008.00438

[B18] Ge X. J. Hsu T. W. Hung K. H. Lin C. J. Huang C. C. Huang C. C. (2012). Inferring multiple refugia and phylogeographical patterns in *Pinus massoniana* based on nucleotide sequence variation and DNA fingerprinting. PloS One 7, e43717. 10.1371/journal.pone.0043717 22952747 PMC3430689

[B19] Guindon S. Gascuel O. (2003). A simple, fast, and accurate algorithm to estimate large phylogenies by maximum likelihood. Syst. Biol. 52, 696–704. 10.1080/10635150390235520 14530136

[B20] Hahn C. Bachmann L. Chevreux B. (2013). Reconstructing mitochondrial genomes directly from genomic next-generation sequencing reads-a baiting and iterative mapping approach. Nucleic Acids Res. 41, e129. 10.1093/nar/gkt371 23661685 PMC3711436

[B21] Hirase S. Takeshima H. Nishida M. Iwasaki W. (2016). Parallel mitogenome sequencing alleviates random rooting effect in phylogeography. Genome Biol. Evol. 8, 1267–1278. 10.1093/gbe/evw063 27016485 PMC4860695

[B22] Jing C. R. Liu H. P. (1999). On the glacial and interglacial stages in quaternary of China. J. Chengdu Univ. Tech. 26, 97–100. 10.3969/j.issn.1671-9727.1999.01.021

[B23] Kimura M. (1980). A simple method for estimating evolutionary rates of base substitutions through comparative studies of nucleotide sequences. J. Mol. Evol. 16, 111–120. 10.1007/BF01731581 7463489

[B24] Kong X. B. Zhang Z. Wang H. B. Wang Y. J. Kong Q. H. (2006). Investigation on sex pheromones in Lasiocampidae: advance and prospect. Sci. Silv. Sin. 6, 115–122. 10.11707/j.1001-7488.20060619

[B25] Li H. Durbin R. (2009). Fast and accurate short read alignment with Burrows-Wheeler transform. Bioinformatics 25, 1754–1760. 10.1093/bioinformatics/btp324 19451168 PMC2705234

[B26] Li J. Qian J. Zhu G. P. Jiang C. Zhang A. B. (2019). Phylogeography of *Dendrolimus punctatus* (Lepidoptera: Lasiocampidae): population differentiation and last glacial maximum survival. Ecol. Evol. 9, 7480–7496. 10.1002/ece3.5278 31346417 PMC6635939

[B27] Li Z. Z. (1991). Monitoring of insecticide-resistance of *Dendrolimus punctatus*. Sci. Silv. Sin. 27, 665–669.

[B28] Librado P. Rozas J. (2009). DnaSP v5: a software for comprehensive analysis of DNA polymorphism data. Bioinformatics 25, 1451–1452. 10.1093/bioinformatics/btp187 19346325

[B29] Louy D. Habel J. C. Schmitt T. Assmann T. Meyer M. Müller P. (2007). Strongly diverging population genetic patterns of three skipper species: the role of habitat fragmentation and dispersal ability. Conserv. Genet. 8, 671–681. 10.1007/s10592-006-9213-y

[B30] Luo R. B. Liu B. H. Xie Y. L. Li Z. Y. Huang W. H. Yuan J. Y. (2012). SOAPdenovo2: an empirically improved memory-efficient short-read *de novo* assembler. GigaScience 1, 18. 10.1186/2047-217X-1-18 23587118 PMC3626529

[B31] Lv K. Wang J. R. Li T. Q. Zhou J. Gu J. Q. Zhou G. X. (2018). Effects of habitat fragmentation on the genetic diversity and differentiation of *Dendrolimus punctatus* (Lepidoptera: Lasiocampidae) in Thousand Island Lake, China, based on mitochondrial *COI* gene sequences. B. Entomol. Res. 109, 1–10. 10.1017/S0007485318000172 29743124

[B32] Ma C. Yang P. Jiang F. Chapuis M. P. Shali Y. Sword G. A. (2012). Mitochondrial genomes reveal the global phylogeography and dispersal routes of the migratory locust. Mol. Ecol. 21, 4344–4358. 10.1111/j.1365-294X.2012.05684.x 22738353

[B33] Mckenna A. Hanna M. Banks E. Sivachenko A. Cibulskis K. Kernytsky A. (2014). The genome analysis toolkit: a mapreduce framework for analyzing next-generation DNA sequencing data. Genome Res. 20, 1297–1303. 10.1101/gr.107524.110 PMC2928508 20644199

[B34] Men Q. L. Xue G. X. Mu D. Hu Q. L. Huang M. Y. (2017). Mitochondrial DNA markers reveal high genetic diversity and strong genetic differentiation in populations of *Dendrolimus kikuchii* Matsumura (Lepidoptera: Lasiocampidae). PloS One 12, 1–16. 10.1371/journal.pone.0179706 PMC5491029 28662066

[B35] Morin P. A. Archer F. I. Foote A. D. Vilstrup J. Allen E. E. Wade P. (2010). Complete mitochondrial genome phylogeographic analysis of killer whales (*Orcinus orca*) indicates multiple species. Genome Res. 20, 908–916. 10.1101/gr.102954.109 20413674 PMC2892092

[B36] Nylander J. A. A. (2004). MrModeltest v2: program distributed by the author (Uppsala: Evolutionary Biology Centre. Uppsala University).

[B37] Papadopoulou A. Anastasiou I. Vogler A. P. (2010). Revisiting the insect mitochondrial molecular clock: the mid-Aegean trench Calibration. Mol. Biol. Evol. 27, 1659–1672. 10.1093/molbev/msq051 20167609

[B38] Peakall R. Lindenmayer D. (2006). Genetic insights into population recovery following experimental perturbation in a fragmented landscape. Biol. Conserv. 132, 520–532. 10.1016/j.biocon.2006.05.013

[B39] Peng L. H. Li Z. Z. (1992). Study on the insecticide-tolerance of *Dendrolimus punctatus* to fenvaleratc. For. Res. 5, 560–564.

[B40] Pritchard J. K. Stephens M. J. Donnelly P. J. (2000). Inference of population structure using multilocus genotype data. Genetics 155, 945–959. 10835412 10.1093/genetics/155.2.945 PMC1461096

[B41] Ronquist F. Huelsenbeck J. P. (2003). MrBayes 3: Bayesian phylogenetic inference under mixed models. Bioinformatics 19, 1572–1574. 10.1093/bioinformatics/btg180 12912839

[B42] Singer M. C. Moore D. R. A. (1991). Genetic variation in oviposition preference between butterfly populations. *J*. Insect Behav. 4, 531–535. 10.1007/BF01049336

[B43] Sumner J. Jessop T. Paetkau D. Moritz C. (2004). Limited effect of anthropogenic habitat fragmentation on molecular diversity in a rain forest skink, *Gnypetoscincus queenslandiae*. Mol. Ecol. 13, 259–269. 10.1046/j.1365-294X.2003.02056.x 14717885

[B44] Sun F. C. Kong X. B. Zhang S. F. Wang H. B. Zhang Z. Liu F. (2017). Geographic variations of sex pheromones in three populations of *Dendrolimus kikuchii* (Lepidoptera: Lasiocampidae). For. Res. 30, 993–998. 10.13275/j.cnki.lykxyj.2017.06.015

[B45] Tajima F. (1989). Statistical method for testing the neutral mutation hypothesis by DNA polymorphism. Genetics 123, 585–595. 2513255 10.1093/genetics/123.3.585 PMC1203831

[B46] Tamura K. Stecher G. Peterson D. Filipski A. Kumar S. (2013). MEGA6: molecular evolutionary genetics analysis version 6.0. Mol. Biol. Evol. 30, 2725–2729. 10.1093/molbev/mst197 24132122 PMC3840312

[B47] Thompson J. D. Gibson T. J. Plewniak F. Jeanmougin F. Higgins D. G. (1997). The CLUSTAL_X Windows interface: flexible strategies for multiple sequence alignment aided by quality analysis tools. Nucleic Acids Res. 25, 4876–4882. 10.1093/nar/25.24.4876 9396791 PMC147148

[B48] Toews D. P. Bresford A. (2012). The biogeography of mitochondrial and nuclear discordance in animals. Mol. Ecol. 21, 3907–3930. 10.1111/j.1365-294X.2012.05664.x 22738314

[B49] Tong Q. He J. Z. (2009). Study on biological characters of *Dendrolimus kikuchii* Matsumura and food consumption of its larva. J. Anhui Agric. Sci. 37, 13122–13124. CNKISUNAHNY.0.2009-27-098

[B50] Wang M. X. Zhou G. Huang X. D. Liao Z. Q. Tong X. W. Xu Z. G. (2008). Research on the effect of insect diversity of the curing *Dendrolimus punctatus* with crop-dusting from airplane. J. Environ. Entomol. 30, 296–300. 10.3724/SP.J.1148.2008.00259

[B51] Wyman S. K. Jansen R. K. Boore J. L. (2004). Automatic annotation of organellar genomes with DOGMA. Bioinformatics 22, 3252–3255. 10.1093/bioinformatics/bth352 15180927

[B52] Xiao G. R. Yan J. J. Xu. C. H. Ding D. M. Shen G. P. Huang C. W. (1964). Studies on the population dynamics of the pine caterpillar (*Dendrolimus punctatus* walker) in China. Sci. Silv. Sin. 9, 201–220.

[B53] Xie Y. Wu G. Tang J. Luo R. Patterson J. Liu S. (2014). SOAPdenovo-Trans: *de novo* transcriptome assembly with short RNA-Seq reads. Bioinformatics 30, 1660–1666. 10.1093/bioinformatics/btu077 24532719

[B54] Xu H. L. Yuan Y. H. He Q. Wu Q. Yan Q. G. Wang Q. (2012). Complete mitochondrial genome sequences of two Chiroptera species (*Rhinolophus luctus* and *Hipposideros armiger*). Mitochondr. DNA 23, 327–328. 10.3109/19401736.2012.674116 22515210

[B55] Yang H. Wang K. (2015). Genomic variant annotation and prioritization with ANNOVAR and wANNOVAR. Nat. Protoc. 10, 1556–1566. 10.1038/nprot.2015.105 26379229 PMC4718734

[B56] You Y. Y. Sun K. P. Xu L. J. Wang L. Jiang T. L. Liu S. (2010). Pleistocene glacial cycle effects on the phylogeography of the Chinese endemic bat species, *Myotis davidii*. BMC Evol. Biol. 10, 208. 10.1186/1471-2148-10-208 20618977 PMC3055248

[B57] Zhang A. B. Li D. M. Chen J. Zhang Z. (2004a). The preliminary study on geohistory of *Dendrolimus punctatus* and its host plants, *Pinus* spp. Entomol. Knowl. 41, 146–149. 10.3969/j.issn.0452-8255.2004.02.011

[B58] Zhang A. B. Kong X. B. Li D. M. Liu Y. Q. (2004b). DNA fingerprinting evidence for the phylogenetic relationship of eight species and subspecies of *Dendrolimus* (Lepidoptera: Lasiocampidae) in China. Acta Entomol. Sin. 47, 236–242. 10.3321/j.issn:0454-6296.2004.02.017

[B59] Zhou Y. F. Abbott R. J. Jiang Z. Y. Du F. K. Milne R. I. Liu J. Q. (2010). Gene flow and species delimitation: a case study of two pine species with overlapping distributions in southeast China. Evolution 64, 2342–2352. 10.1111/j.1558-5646.2010.00988.x 20298431

