# Supplementary material for: Lineage Divergence of Dendrolimus punctatus in Southern China Based on Mitochondrial Genome
Source: Front Genet. 2020 Feb 19;11:65. doi: 10.3389/fgene.2020.00065 (PMC7045034; doi:10.3389/fgene.2020.00065)
Supplement: Table S1 — Sampling information of Dendrolimus punctatus in China. [file Table_1.docx]

**Table S1**. Sampling information of *Dendrolimus punctatus* in China

| Province | Location | Population code | Geographic coordinate | | Sample size | GenBank accession numbers of Mitochondrial genomes |
| --- | --- | --- | --- | --- | --- | --- |
|  |  |  | Latitude(N) | Longitude(W) |  |  |
| Anhui | Hefei | AHHF | 31°82’N | 117°23’E | 4 | MN605223;MN605224;MN864178;MN864179 |
| Chongqing | Nanchuan | CQNC | 29°15’N | 107°07’E | 3 | MN864168;MN864169;MN758738 |
|  | Yubei | CQYB | 29°71’N | 106°63’E | 3 | MN788467;MN864166;MN864167 |
| Guangdong | Luoding | GDLD | 22°77’N | 111°57’E | 4 | MN605222;MN864176;MN864177;MN758739 |
| Guangxi | Baise | GXBS | 23°90’N | 106°61’E | 3 | MN605220;MN864173;MN864174 |
|  | Guilin | GXGL | 25°27’N | 110°29’E | 3 | MN758737; MN605221;MN864175 |
| Guizhou | Jinping | GZJP | 26°67’N | 109°20’E | 4 | MN788468;MN864170;MN864171;MN864172 |
| Hubei | Dawu | HBDW | 31°57’N | 114°12’E | 3 | MN605211;MN864159;MN864160 |
|  | Huanggang | HBHG | 31°28’N | 114°62’E | 3 | MN864161;MN861162;MN864163 |
| Hunan | Chenzhou | HNCZ | 25°78’N | 113°02’E | 3 | MN605212; MN605213;MN864164 |
|  | Dongan | HNDA | 26°40’N | 111°31’E | 3 | MN605226; MN605214;MN864165 |
| Jiangxi | Xingguo | JXXG | 26°34’N | 115°36’E | 3 | MN605225; MN605218; MN605219 |
|  | Yudu | JXYD | 25°95’N | 115°41’E | 3 | MK886799; MN758735;MN758734 |
| Sichuan | Dazhou | SCDZ | 30°73’N | 107°20’E | 3 | MN788465;MN605215; MN605216 |
|  | Yibin | SCYB | 28°75’N | 104°64’E | 3 | MN788466; MN758736; MN605217 |
