# Supplementary figures and images for: Lineage Divergence of Dendrolimus punctatus in Southern China Based on Mitochondrial Genome
Source: Front Genet. 2020 Feb 19;11:65. doi: 10.3389/fgene.2020.00065 (PMC7045034; doi:10.3389/fgene.2020.00065)

**Fig. S1** Population structure branch optimum value analysis


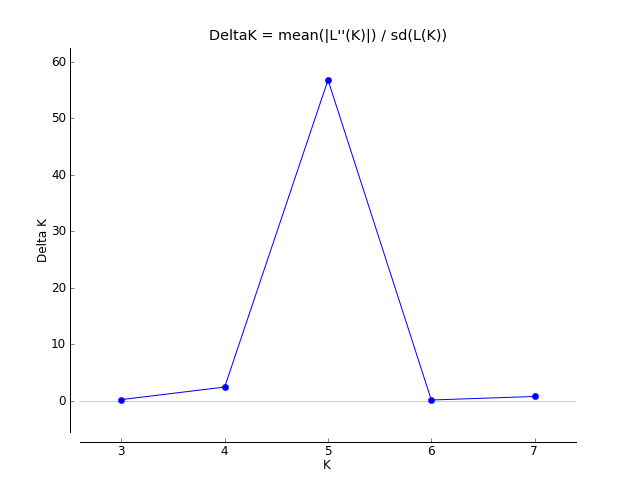

Supplement: Figure S1 — Population structure branch optimum value analysis. [file DataSheet_1.docx]
